# Announcement

**DOI:** 10.1155/S1463924600000389

**Published:** 2000


					Announcement
Twenty- rst Annual Analytical Chemistry Starter
Grant Award
The Society for Analytical Chemists of Pittsburgh awards
one grant annually of $20 000 to an assistant professor in
the (R) eld of analytical chemistry. The purpose of this
grant is to encourage high-quality, innovative research
by a new analytical chemistry professor, and to promote
the training and development of graduate students in this
(R) eld. Assistant professors who have accepted a United
States college or university appointment since 31 Decem-
ber 1996 are eligible.
The award for the year 2000 has been made to Dr Aaron
Timperman of the Eberly College of Arts & Sciences,
Department of Chemistry, West Virginia University,
Morgantown, WV 26506, USA.
For more information, please contact James Chadwick, Chairman,
Starter Grant Committee, Society for Analytical Chemists
of Pittsburgh, 300 Penn Center Blvd., Ste. 332, Pittsburgh, PA
15234, USA. Tel: 1 1 800 825 3221, Extn 208; Fax: 1 1 412
825 3224
Journal of Automated Methods & Management in Chemistry, Vol. 22, No. 6 (November-December 2000) p. 207
Journal of Automated Methods & Management in Chemistry ISSN 1463 9246 print/ISSN 1464 5068 online # 2000 Taylor & Francis Ltd
http://www.tandf.co.uk/journals
207

				

